# 1-(2,4-Difluoro­phen­yl)-2-(1*H*-1,2,4-triazol-1-yl)ethanol

**DOI:** 10.1107/S1600536812020661

**Published:** 2012-05-16

**Authors:** Victor Kesternich, Ronald Nelson-González, Marcia Pérez-Fehrmann, Alejandro Cárdenas, Iván Brito

**Affiliations:** a Departamento de Química, Universidad Católica del Norte, Casilla 1280, Antofagasta, Chile; b Departamento de Física, Facultad de Ciencias Básicas, Universidad de Antofagasta, Casilla 170, Antofagasta, Chile; c Departamento de Química, Facultad de Ciencias Básicas, Universidad de Antofagasta, Casilla 170, Antofagasta, Chile

## Abstract

In the title compound, C_10_H_9_F_2_N_3_O, the dihedral angle between the mean planes of the triazole and benzene rings is 20.6 (2)°. In the crystal, mol­ecules are linked by strong O—H⋯ N hydrogen bonds into chains with graph-set notation *C*(9) along [100]. Weak C—H⋯N and C—H⋯F inter­actions are also observed.

## Related literature

For phenacyl­azole derivatives, see: Emami *et al.* (2008 ▶, 2009 ▶). For their biological properties, see: Schiaffella *et al.* (2005 ▶). For graph-set notation, see: Bernstein *et al.* (1995 ▶).
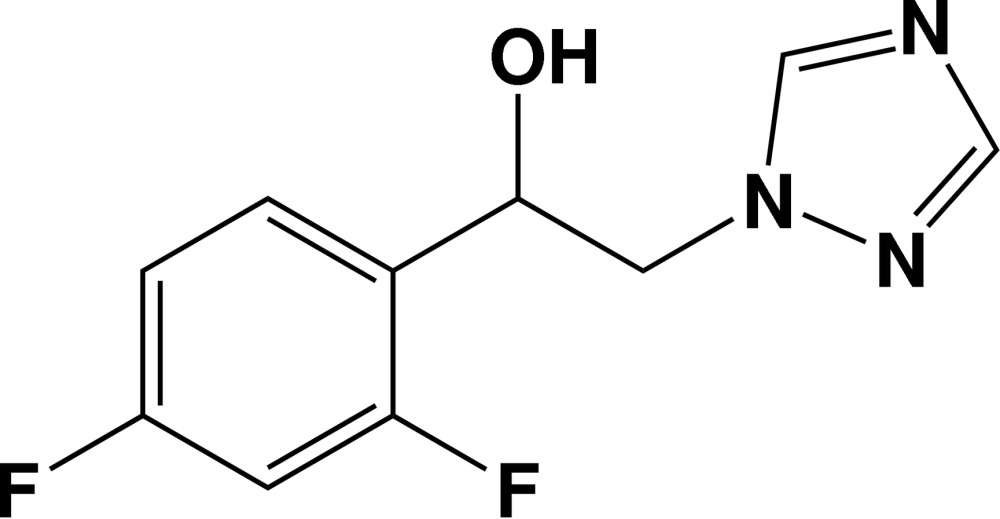


## Experimental

### 

#### Crystal data

C_10_H_9_F_2_N_3_O
*M* *_r_* = 225.20
Orthorhombic, 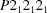

*a* = 5.3770 (11) Å
*b* = 12.598 (3) Å
*c* = 15.601 (3) Å
*V* = 1056.8 (3) Å^3^
*Z* = 4
Mo *K*α radiation
μ = 0.12 mm^−1^
*T* = 295 K
0.60 × 0.29 × 0.08 mm

#### Data collection

Nonius KappaCCD area-detector diffractometer
9658 measured reflections
1536 independent reflections
1344 reflections with *I* > 2σ(*I*)
*R*_int_ = 0.081

#### Refinement

*R*[*F* ^2^ > 2σ(*F* ^2^)] = 0.063
*wR*(*F* ^2^) = 0.149
*S* = 1.16
1536 reflections
146 parameters
H-atom parameters constrained
Δρ_max_ = 0.16 e Å^−3^
Δρ_min_ = −0.16 e Å^−3^

### 

Data collection: *COLLECT* (Nonius, 2000 ▶); cell refinement: *DENZO-SMN* (Otwinowski & Minor, 1997 ▶); data reduction: *DENZO-SMN*; program(s) used to solve structure: *SHELXS97* (Sheldrick, 2008 ▶); program(s) used to refine structure: *SHELXL97* (Sheldrick, 2008 ▶); molecular graphics: *OLEX2* (Dolomanov *et al.*, 2009 ▶); software used to prepare material for publication: *WinGX* (Farrugia, 1999 ▶) and *publCIF* (Westrip, 2010 ▶).

## Supplementary Material

Crystal structure: contains datablock(s) I, global. DOI: 10.1107/S1600536812020661/su2423sup1.cif

Structure factors: contains datablock(s) I. DOI: 10.1107/S1600536812020661/su2423Isup2.hkl

Supplementary material file. DOI: 10.1107/S1600536812020661/su2423Isup3.cml

Additional supplementary materials:  crystallographic information; 3D view; checkCIF report

## Figures and Tables

**Table 1:** Hydrogen-bond geometry (Å, °)

*D*—H⋯*A*	*D*—H	H⋯*A*	*D*⋯*A*	*D*—H⋯*A*
O1—H1⋯N3^i^	0.82	2.01	2.826 (4)	171
C3—H3⋯N2^ii^	0.93	2.62	3.500 (5)	158
C8—H8*B*⋯F1^iii^	0.97	2.45	3.340 (5)	153
C10—H10⋯F2^iv^	0.93	2.48	3.270 (4)	142

Symmetry codes: (i) 
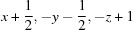
; (ii) 
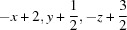
; (iii) 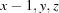; (iv) 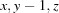.

**Fig. 1.**
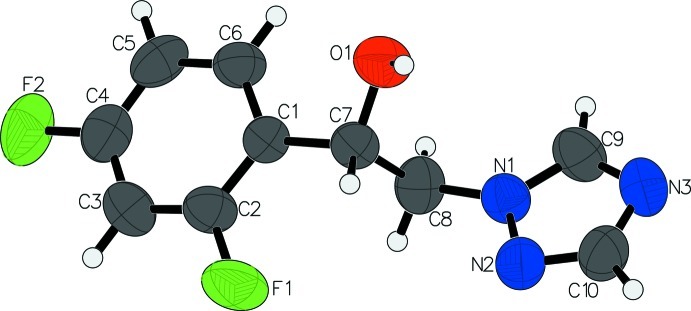
A view of the molecular structure of the title molecule, with the atom numbering. Displacement ellipsoids are drawn at the 50% probability level.

**Fig. 2.**
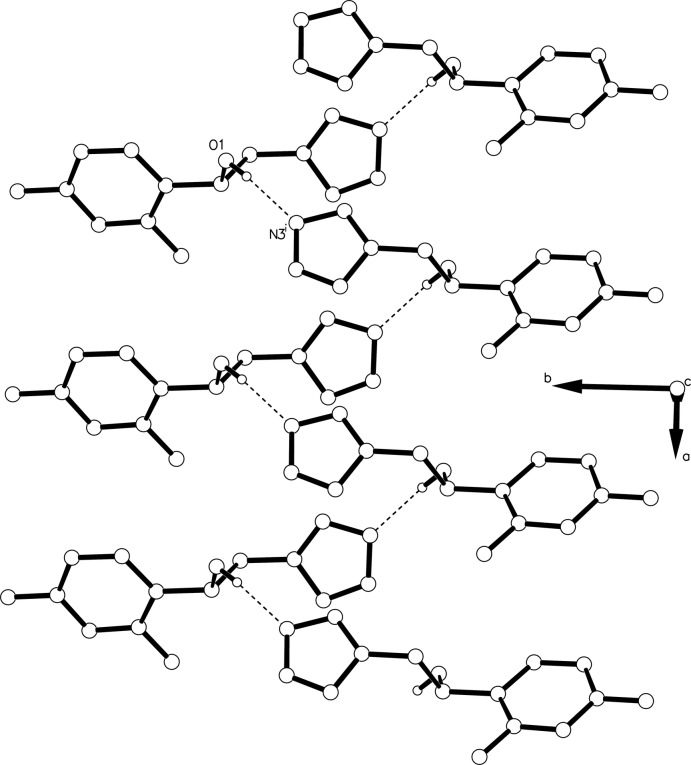
A partial view along the c axis of the crystal packing of the title compound, showing the formation of the O—H··· N hydrogen bonded chain [dashed lines; see Table 1 for details; the H-atoms not involved in hydrogen-bonding have been omitted for clarity]

## supplementary crystallographic information

### Comment

Phenacyl azole derivatives (Emami *et al.*, 2008; Emami *et al.*, 2009) are very important due to their antifungal properties (Schiaffella *et al.*, 2005). We report herein on the synthesis and crystal structure of the title compound, a member of this important family of compounds.

In the title molecule, Fig. 1, the dihedral angle between the mean planes of the triazole and benzene rings is 20.6 (2)°.

In the crystal, molecules are linked by strong O—H··· N hydrogen bonds (Fig. 2 and Table 1) into chains propagating along [100] which have a C(9) graph-set notation (Bernstein *et al.*, 1995). There are also weak C-H···N and C-H···F interactions present (Table 1).

### Experimental

To a stirred solution of 1-(2,4-difluorophenyl)-2-(1*H*-1,2,4-triazol-1-yl)ethanone (6.00 g, 26.88 mmol) in methanol (50 ml), sodium borohydride (1.12 g, 29.57 mmol) dissolved in methanol (20 ml), was added drop wise. The reaction mixture was then stirred at room temperature for 30 min. After completion of the reaction, the solvent was removed under vacuum, and 25 ml of cold water was added. Extraction was performed with dichloromethane (3 × 40 ml), and the organic extracts were washed with water (3 × 30 ml). The organic phase was then dried over anhydrous sodium sulfate. After evaporation of the solvent under vacuum, the residue was purified by crystallization in ethanol to provide the title compound as colourless crystals [85% yield; M.p.: 391-393 K].

### Refinement

All of the H atoms could be located in difference Fourier maps. In the final cycles of refinement they were included in calculated positions and treated as riding atoms: O-H = 0.82 Å, C—H = 0.93 and 0.97 Å for CH and CH_2_ H atoms, respectively, with *U*_iso_(H) = k × *U*_eq_(O,C), where k = 1.5 for the OH H atom, and = 1.2 for other H atoms. In the final cycles of refinement, in the absence of significant anomalous scattering effects, 1126 Friedel pairs were merged and Δf " set to zero.

### Crystal data

**Table:**

C_10_H_9_F_2_N_3_O	*F*(000) = 464
*M**_r_* = 225.20	*D*_x_ = 1.415 Mg m^−^^3^
Orthorhombic, *P*2_1_2_1_2_1_	Mo *K*α radiation, λ = 0.71073 Å
*a* = 5.3770 (11) Å	µ = 0.12 mm^−^^1^
*b* = 12.598 (3) Å	*T* = 295 K
*c* = 15.601 (3) Å	Plate, colourless
*V* = 1056.8 (3) Å^3^	0.60 × 0.29 × 0.08 mm
*Z* = 4	

### Data collection

**Table:**

Nonius KappaCCD area-detector diffractometer	1344 reflections with *I* > 2σ(*I*)
Radiation source: fine-focus sealed tube	*R*_int_ = 0.081
Graphite monochromator	θ_max_ = 28.7°, θ_min_ = 4.2°
φ and ω scans with κ offsets	*h* = 0→7
9658 measured reflections	*k* = 0→16
1536 independent reflections	*l* = 0→21

### Refinement

**Table:**

Refinement on *F*^2^	Primary atom site location: structure-invariant direct methods
Least-squares matrix: full	Secondary atom site location: difference Fourier map
*R*[*F*^2^ > 2σ(*F*^2^)] = 0.063	Hydrogen site location: inferred from neighbouring sites
*wR*(*F*^2^) = 0.149	H-atom parameters constrained
*S* = 1.16	*w* = 1/[σ^2^(*F*_o_^2^) + (0.0491*P*)^2^ + 0.3301*P*] where *P* = (*F*_o_^2^ + 2*F*_c_^2^)/3
1536 reflections	(Δ/σ)_max_ < 0.001
146 parameters	Δρ_max_ = 0.16 e Å^−^^3^
0 restraints	Δρ_min_ = −0.16 e Å^−^^3^

### Special details

**Table:**

Geometry. Bond distances, angles etc. have been calculated using the rounded fractional coordinates. All su's are estimated from the variances of the (full) variance-covariance matrix. The cell esds are taken into account in the estimation of distances, angles and torsion angles
Refinement. Refinement of *F*^2^ against ALL reflections. The weighted *R*-factor *wR* and goodness of fit *S* are based on *F*^2^, conventional *R*-factors *R* are based on *F*, with *F* set to zero for negative *F*^2^. The threshold expression of *F*^2^ > 2σ(*F*^2^) is used only for calculating *R*-factors(gt) *etc*. and is not relevant to the choice of reflections for refinement. *R*-factors based on *F*^2^ are statistically about twice as large as those based on *F*, and *R*- factors based on ALL data will be even larger.

### Fractional atomic coordinates and isotropic or equivalent isotropic displacement parameters (Å^2^)

**Table:**

	*x*	*y*	*z*	*U*_iso_*/*U*_eq_	
F1	1.0193 (5)	0.0759 (2)	0.69521 (16)	0.0959 (9)	
F2	0.7227 (6)	0.42266 (16)	0.67885 (15)	0.1001 (12)	
O1	0.5799 (5)	−0.0141 (2)	0.49259 (14)	0.0733 (9)	
N1	0.5125 (5)	−0.1766 (2)	0.62303 (17)	0.0583 (8)	
N2	0.7087 (6)	−0.2399 (2)	0.6393 (2)	0.0725 (10)	
N3	0.3927 (6)	−0.3347 (3)	0.5866 (2)	0.0716 (11)	
C1	0.6906 (6)	0.1114 (2)	0.60153 (17)	0.0491 (8)	
C2	0.8583 (6)	0.1469 (3)	0.6608 (2)	0.0615 (10)	
C3	0.8761 (8)	0.2503 (3)	0.6881 (2)	0.0707 (11)	
C4	0.7107 (8)	0.3195 (3)	0.6541 (2)	0.0686 (13)	
C5	0.5350 (8)	0.2905 (3)	0.5961 (2)	0.0703 (13)	
C6	0.5262 (7)	0.1855 (3)	0.5700 (2)	0.0614 (10)	
C7	0.6804 (6)	−0.0045 (2)	0.57500 (18)	0.0528 (9)	
C8	0.5227 (7)	−0.0634 (3)	0.6398 (2)	0.0655 (11)	
C9	0.3289 (7)	−0.2346 (3)	0.5921 (2)	0.0699 (11)	
C10	0.6262 (8)	−0.3331 (3)	0.6162 (3)	0.0744 (14)	
H1	0.66000	−0.05800	0.46520	0.1100*	
H3	0.99530	0.27150	0.72770	0.0850*	
H5	0.42310	0.34000	0.57450	0.0840*	
H6	0.40680	0.16460	0.53030	0.0740*	
H7	0.84910	−0.03400	0.57530	0.0630*	
H8A	0.59030	−0.05180	0.69660	0.0790*	
H8B	0.35530	−0.03490	0.63880	0.0790*	
H9	0.17440	−0.20790	0.57620	0.0840*	
H10	0.72300	−0.39410	0.62000	0.0900*	

### Atomic displacement parameters (Å^2^)

**Table:**

	*U*^11^	*U*^22^	*U*^33^	*U*^12^	*U*^13^	*U*^23^
F1	0.0860 (16)	0.0923 (16)	0.1095 (16)	0.0289 (14)	−0.0421 (15)	−0.0148 (14)
F2	0.157 (3)	0.0540 (11)	0.0894 (14)	−0.0013 (15)	0.0206 (18)	−0.0154 (10)
O1	0.0848 (18)	0.0700 (15)	0.0650 (12)	0.0173 (14)	−0.0112 (13)	−0.0159 (11)
N1	0.0499 (13)	0.0594 (14)	0.0656 (14)	0.0022 (13)	−0.0011 (13)	−0.0030 (12)
N2	0.0575 (17)	0.0571 (16)	0.103 (2)	0.0034 (13)	−0.0163 (18)	0.0053 (15)
N3	0.0719 (19)	0.0660 (18)	0.0770 (18)	−0.0127 (16)	0.0007 (16)	−0.0090 (14)
C1	0.0462 (15)	0.0494 (14)	0.0517 (13)	0.0050 (12)	0.0040 (12)	−0.0018 (12)
C2	0.0571 (18)	0.0657 (19)	0.0616 (17)	0.0112 (16)	−0.0070 (15)	−0.0052 (15)
C3	0.072 (2)	0.075 (2)	0.0650 (18)	−0.0056 (19)	−0.0057 (19)	−0.0142 (17)
C4	0.091 (3)	0.0569 (17)	0.0578 (16)	0.001 (2)	0.016 (2)	−0.0059 (14)
C5	0.085 (3)	0.0578 (18)	0.0682 (19)	0.0215 (19)	0.007 (2)	0.0092 (15)
C6	0.0638 (19)	0.0613 (17)	0.0590 (16)	0.0096 (17)	−0.0086 (16)	−0.0008 (14)
C7	0.0481 (15)	0.0521 (15)	0.0581 (15)	0.0081 (14)	−0.0015 (14)	−0.0054 (13)
C8	0.0637 (19)	0.0569 (17)	0.076 (2)	0.0040 (17)	0.0088 (18)	−0.0080 (15)
C9	0.0487 (17)	0.080 (2)	0.081 (2)	−0.0026 (18)	−0.0050 (17)	−0.0055 (19)
C10	0.070 (2)	0.0563 (19)	0.097 (3)	0.0018 (18)	−0.008 (2)	0.0063 (18)

### Geometric parameters (Å, º)

**Table:**

F1—C2	1.356 (4)	C2—C3	1.374 (5)
F2—C4	1.357 (4)	C3—C4	1.354 (6)
O1—C7	1.400 (4)	C4—C5	1.358 (5)
O1—H1	0.8200	C5—C6	1.385 (5)
N1—C8	1.451 (5)	C7—C8	1.514 (5)
N1—C9	1.320 (5)	C3—H3	0.9300
N1—N2	1.347 (4)	C5—H5	0.9300
N2—C10	1.306 (5)	C6—H6	0.9300
N3—C10	1.338 (5)	C7—H7	0.9800
N3—C9	1.310 (5)	C8—H8A	0.9700
C1—C2	1.367 (4)	C8—H8B	0.9700
C1—C7	1.519 (4)	C9—H9	0.9300
C1—C6	1.377 (5)	C10—H10	0.9300
			
C7—O1—H1	109.00	N1—C8—C7	112.5 (3)
N2—N1—C8	121.2 (3)	N1—C9—N3	111.2 (3)
C8—N1—C9	129.7 (3)	N2—C10—N3	115.3 (4)
N2—N1—C9	109.1 (3)	C2—C3—H3	122.00
N1—N2—C10	102.4 (3)	C4—C3—H3	122.00
C9—N3—C10	102.1 (3)	C4—C5—H5	121.00
C2—C1—C7	121.5 (3)	C6—C5—H5	121.00
C6—C1—C7	122.1 (3)	C1—C6—H6	119.00
C2—C1—C6	116.3 (3)	C5—C6—H6	119.00
F1—C2—C3	117.3 (3)	O1—C7—H7	109.00
C1—C2—C3	124.5 (3)	C1—C7—H7	109.00
F1—C2—C1	118.3 (3)	C8—C7—H7	109.00
C2—C3—C4	116.3 (3)	N1—C8—H8A	109.00
F2—C4—C3	118.3 (3)	N1—C8—H8B	109.00
C3—C4—C5	123.0 (4)	C7—C8—H8A	109.00
F2—C4—C5	118.7 (3)	C7—C8—H8B	109.00
C4—C5—C6	118.5 (4)	H8A—C8—H8B	108.00
C1—C6—C5	121.4 (3)	N1—C9—H9	124.00
O1—C7—C8	110.8 (3)	N3—C9—H9	124.00
C1—C7—C8	108.0 (2)	N2—C10—H10	122.00
O1—C7—C1	110.3 (2)	N3—C10—H10	122.00
			
C8—N1—N2—C10	179.9 (3)	C7—C1—C6—C5	178.8 (3)
C9—N1—N2—C10	0.1 (4)	C2—C1—C7—O1	−154.7 (3)
N2—N1—C8—C7	73.8 (4)	C2—C1—C7—C8	84.1 (4)
C9—N1—C8—C7	−106.5 (4)	C6—C1—C7—O1	27.9 (4)
N2—N1—C9—N3	−0.1 (4)	C6—C1—C7—C8	−93.3 (3)
C8—N1—C9—N3	−179.9 (3)	F1—C2—C3—C4	−178.8 (3)
N1—N2—C10—N3	−0.1 (5)	C1—C2—C3—C4	1.3 (5)
C10—N3—C9—N1	0.0 (4)	C2—C3—C4—F2	−179.6 (3)
C9—N3—C10—N2	0.1 (5)	C2—C3—C4—C5	0.0 (6)
C6—C1—C2—F1	178.3 (3)	F2—C4—C5—C6	179.0 (3)
C6—C1—C2—C3	−1.9 (5)	C3—C4—C5—C6	−0.6 (6)
C7—C1—C2—F1	0.7 (4)	C4—C5—C6—C1	0.0 (5)
C7—C1—C2—C3	−179.5 (3)	O1—C7—C8—N1	62.4 (3)
C2—C1—C6—C5	1.2 (5)	C1—C7—C8—N1	−176.7 (3)

### Hydrogen-bond geometry (Å, º)

**Table:**

*D*—H···*A*	*D*—H	H···*A*	*D*···*A*	*D*—H···*A*
O1—H1···N3^i^	0.82	2.01	2.826 (4)	171
C3—H3···N2^ii^	0.93	2.62	3.500 (5)	158
C8—H8*B*···F1^iii^	0.97	2.45	3.340 (5)	153
C10—H10···F2^iv^	0.93	2.48	3.270 (4)	142

Symmetry codes: (i) *x*+1/2, −*y*−1/2, −*z*+1; (ii) −*x*+2, *y*+1/2, −*z*+3/2; (iii) *x*−1, *y*, *z*; (iv) *x*, *y*−1, *z*.
